# Does orthodontic space opening in patients with congenitally missing maxillary lateral incisors also reduce the need for bone grafting during implant placement? A retrospective study

**DOI:** 10.34172/japid.025.3765

**Published:** 2025-10-22

**Authors:** Maryam Omidkhoda, Seyed Hosein Hoseini Zarch, Arezoo Jahanbin, Parisa Hatami, Alireza Ghasemzadeh

**Affiliations:** ^1^Department of Orthodontics, School of Dentistry, Mashhad University of Medical Sciences, Mashhad, Iran; ^2^Dental Research Center, School of Dentistry, Mashhad University of Medical Sciences, Mashhad, Iran; ^3^Department of Orthodontics, School of Dentistry, Yazd University of Medical Sciences, Yazd, Iran

**Keywords:** Alveolar bone atrophy, Cone-beam computed tomography, Dental implants, Tooth abnormalities

## Abstract

**Background.:**

Different studies have provided inconsistent results regarding the effectiveness of orthodontic tooth movement in establishing an adequate width and height of the edentulous ridge in patients with missing maxillary lateral incisors. This study aimed to compare the dimensions and density of the alveolar ridge after canine distalization for the preparation of implant placement and after no significant canine movement along the ridge.

**Methods.:**

Sixteen patients (30 sites) with congenitally missing teeth were included in this retrospective study. The patients were divided into two groups: group 1: patients with erupted canines adjacent to the central incisor treated for canine distalization; group 2: patients with erupted canine almost in the correct position, treated with canine alignment. The alveolar ridge width, height, buccal undercut, and density were measured by cone-beam computed tomography (CBCT). The data were analyzed according to sex, age, and type of orthodontic treatment. Chi-square test, t-test, and Pearson’s correlation were used. The significance level was 0.05.

**Results.:**

No significant differences were found between the two groups in alveolar ridge width at 3 mm and 6 mm apical to the alveolar crest, height, buccal undercut depth, and density in the position of the missing lateral incisors (*P*>0.05).

**Conclusion.:**

Movement of the canine along the alveolar ridge in patients with congenitally missing maxillary lateral incisors did not significantly affect alveolar ridge width, height, buccal undercut, and density. Therefore, the effectiveness of canine distalization treatment in reducing the need for bone grafting is questionable.

## Introduction

 Upper lateral incisors are the second most common missing teeth in adults, after the lower second premolars.^1^ Different populations have significantly different frequencies of congenitally absent maxillary lateral incisors; however, most reports in the literature show a range between 1% and 3% for missing lateral incisors.^2^ Missing lateral incisors cause problems such as the unpleasant appearance of the patient’s smile, deviation of the dental midline, and asymmetry of the dental arch, making it necessary to perform therapeutic intervention. Generally, two types of treatments are offered for this problem: opening the space and placing a dental prosthesis and implant or closing the space by bringing the canine tooth forward and reshaping it as a lateral tooth. The choice between these two is based on the type of malocclusion, the patient’s profile, and the size, shape, and color of the canine.^3^

 After considering all the conditions, if the patient’s treatment plan entails opening the space for implant placement, it should be ensured that enough bone is present in the toothless area. Bone grafting is necessary if the width or height of the edentulous ridge is inadequate. Several authors have suggested that, as an alternative to bone grafting, orthodontic movement of the adjacent canine tooth along the defective alveolar ridge can be useful for creating sufficient bone in the edentulous site. This is especially true when the canine erupts near the central incisor and is distalized by orthodontic force to create space for the missing lateral implant.^4,5^

 There is inconsistency in the literature regarding the effectiveness of orthodontic tooth movement in establishing an adequate buccolingual width and vertical height of the edentulous ridge. Several investigators, such as Beyer et al^6^ and Uribe et al,^7,8^ have concluded that a significant volume deficiency exists immediately after orthodontic tooth movement at the site of the missing lateral incisor. In contrast, Nováčková et al^9^ found that the ridge of the maxillary lateral incisor is well preserved in the short and long term, with insignificant clinical losses in width and height immediately after ridge development through orthodontic tooth movement. Moreover, most research in this field has used plaster models to evaluate the changes made in the alveolar ridge, although these casts cannot accurately show the changes that have occurred in the underlying bone. On the other hand, our search in the available databases showed that no studies have compared the dimensions and density of the alveolar ridge at the location of missing lateral teeth between the two groups with and without canine tooth distalization.

 Therefore, this study aimed to determine and compare the dimensions and density of the alveolar ridge using cone-beam computed tomography (CBCT) in patients with congenitally missing maxillary lateral incisors between two groups with and without distalization of the canine.

 The null hypothesis: There is no difference in the dimensions and density of the alveolar ridge between patients who underwent distalization of the canine and those who did not.

## Methods

 The study protocol of the present retrospective radiographic study was approved by the Ethics Committee of Mashhad University of Medical Science (IR.MUMS.DENTISTRY.REC.1400.038).CBCT scans of patients with congenitally missing maxillary lateral incisor who were referred for placement of missing tooth implants were collected from a private maxillofacial radiology center in Mashhad.

 The inclusion criteria were patients 15‒38 years of age, unilateral or bilateral congenitally missing maxillary lateral incisor, receiving orthodontic treatment to open the space or align the teeth (in case of sufficient space between the central incisor and canine) in the candidate to receive an implant in the location of the missing lateral tooth, and presence of CBCT scan after orthodontic treatment and before implant placement. The exclusion criteria were the presence of a deciduous lateral incisor, an impacted or completely unerupted permanent canine, cleft palate, or any other dentofacial deformity; patients undergoing orthodontic treatment to close the space and substituting the missing lateral incisor with the canine; and patients with systemic bone disease or a history of periodontal disease.

 Records of a private oral and maxillofacial radiology center over two years (2021-2022) were screened to identify patients with congenitally missing maxillary lateral incisors who were referred for implant placement in the region of the missing tooth. The CBCT scan had to be performed in the presence of brackets in the patient’s mouth or less than three months after the end of orthodontic treatment. All of the CBCT images were acquired using a Planmeca Viso G7 scanner (Planmeca, Helsinki, Finland) with a 90 × 90-mm field-of-view (FOV), 200-mm voxel size, and the following scan parameters: 90 kVp tube voltage, 9 mA tube current, and 12-second scan time. Planmeca Romexis (5.3.4.39) software was used to analyze the prepared scans. The same assessor performed all the measurements to prevent inter-examiner error. Finally, only 16 patient records met the inclusion criteria for the current study.

 The final sample consisted of two patients with unilateral maxillary lateral incisor agenesis and 14 patients with bilateral maxillary lateral incisor agenesis (30 missing teeth), which included nine women and seven men with an average age of 25 years. Patient information was collected from the respective orthodontic centers and recorded on a checklist. These data included age, sex, and the type of orthodontic treatment based on canine tooth movement (canine distalization or just alignment).

 The patients were divided into two groups based on the type of orthodontic treatment. In the first group, the canine tooth had erupted in the vicinity of the central tooth, and more than half of the missing lateral incisor tooth width along the ridge was distalized ( > 3 mm). In the second group, the canine tooth had erupted almost in its original place, and less than half of the lateral incisor width along the ridge was distalized. Its orthodontic treatment mainly consisted of aligning the teeth. It should also be mentioned that some patients had a wide diastema, or in other words, two central incisors were distally positioned, and their orthodontic treatment mainly included the mesial movement of the two central teeth. Moreover, these patients were also considered as part of the first group because the central teeth were moved along the alveolar ridge, and their effect was similar to that of canine tooth movement along the alveolar ridge. All cases were treated with the 022 MBT system. Canine distalization in the first group was performed primarily using an open coil and, if necessary, with chain and elastic, using an 0.018-inch base archwire. Alignment and movement of the canine in the non-distalization group was performed using orthodontic wire. Attempt was made to maintain the correct axial inclination of canine during distalization and the movement was mainly of the bodily type.

**Figure 1:**
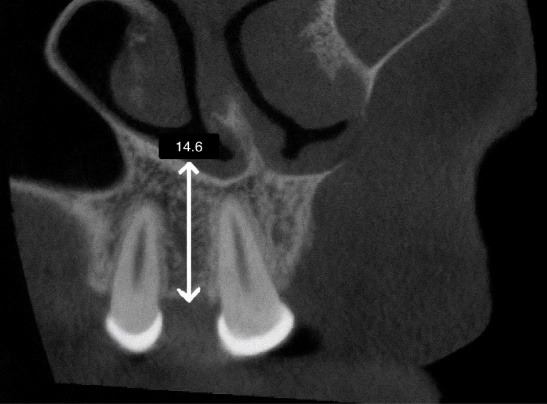
Alveolar ridge height measurement using CBCT at the site of lateral incisor agenesis with 14.6 mm of ridge height in this patient

 To measure the alveolar ridge height in CBCT scans, the deepest part of the alveolar crest ridge to the line connecting the cementoenamel junctions of the maxillary canine and central incisor was determined on the coronal slice. Height measurements were made from the deepest point to the floor of the nose (Figure 1).

**Figure 2:**
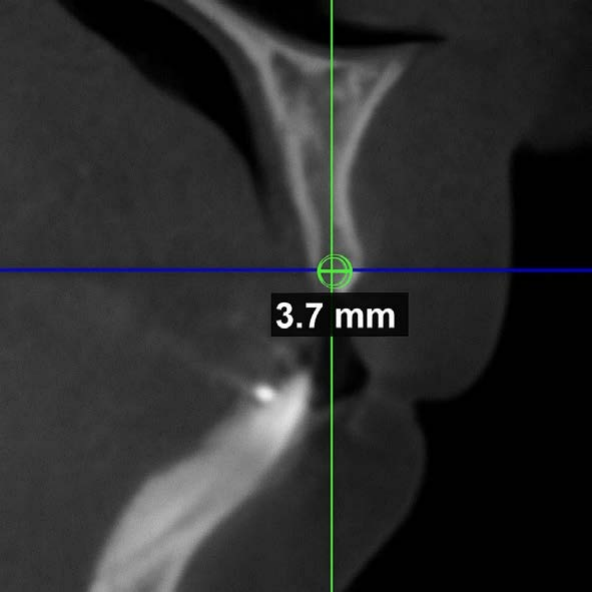
Alveolar bone width measurements using CBCT at the site of lateral incisor agenesis at 3 mm from the edge of the alveolar crest with 3.7 mm of ridge width in this patient

 Alveolar bone width measurements in CBCT scans were performed along the sagittal reference plane at 3 mm and 6 mm apical to the alveolar bone crest. In other words, the buccolingual width of the alveolar ridge was measured in the sagittal slice at 3 and 6 mm from the deepest point of the alveolar crest in the edentulous region (Figure 2).

**Figure 3:**
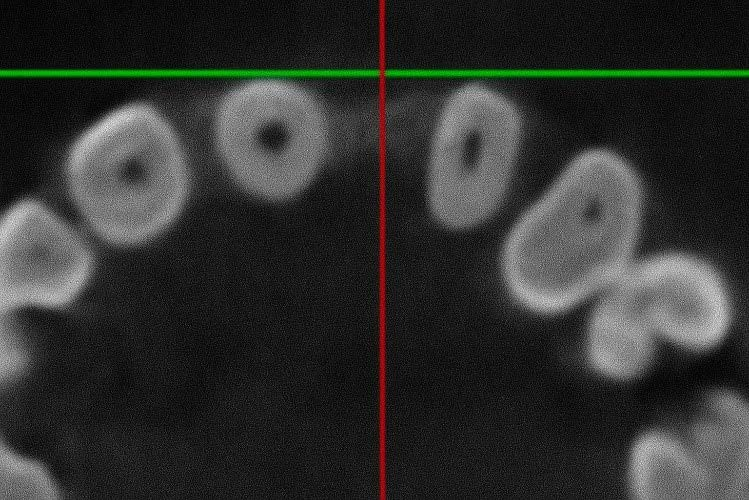
Buccal undercut depth measurement from axial CBCT slice at the site of lateral incisor agenesis

 To measure the depth of the buccal undercut, first, in the three-dimensional scan, the deepest point of the undercut was found around the connecting line of the alveolar crest. Then, in the occlusal (axial) cut, a tangent to the buccal cortical plane was drawn on both sides of the concave area, parallel to the main axis of the alveolar ridge. Finally, the depth of labial concavity was measured from the deepest point of the undercut to this line (Figure 3).

 Concerning bone density, the Hounsfield units (HU) of the implant placement area was measured using Planmeca Romexis (5.3.4.39) software.

 By comparing the two means with a 95% confidence level and 95% power, and according to the article by Uribe et al,^7^ the sample size in each group was calculated at 9 missing teeth, but for more certainty and ease of access to more samples, this number increased to 10 missing teeth in each group.

 Data were collected and analyzed using SPSS 16.0 (SPSS Inc., Chicago, IL, USA). Means, standard deviations, and maximum and minimum values were reported for all variables. Since the data were normally distributed according to the results of the Kolmogorov-Smirnov test, the independent t-test was used for data analysis and to compare the results. Pearson’s correlation coefficient was used to evaluate the correlation between the studied variables and age. Statistical significance was set at *P* < 0.05.

## Results

Table 1 shows the number and percentage of males and females in each of the two groups with and without canine distalization. The mean orthodontic treatment time in the canine distalization group was 3 years and 4 months, with 2 years and 3 months in the non-distalization group. The results of the chi-squared test showed no significant difference in the sex distribution between the two study groups (*P* = 0.79).

Table 2 reports the mean, minimum, maximum, standard deviation values, and significance of the investigated variables, including age, height of the alveolar ridge, width of the ridge at 3 mm and 6 mm from the edge of the alveolar crest, and density and depth of the labial undercut according to the treatment groups. The results indicated that the average age in the canine distalization treatment group was 0.9 years more than the non-distalization treatment group (*P* = 0.76). Also, in the group with canine distalization treatment, the average height of the alveolar ridge and the average width of the ridge at 3 mm from the edge of the alveolar crest were 0.2 mm (*P* = 0.83), and 0.39 mm (*P* = 0.31) more than the group with non-distalization treatment, respectively. In the group with non-distalization treatment, the average width of the ridge at 6 mm from the edge of the alveolar crest and the average depth of the buccal undercut were 0.28 mm (*P* = 0.59) and 0.14 mm (*P* = 0.53) more than the group with distalization treatment, respectively. In general, the statistical analysis did not show any statistically significant difference between the distalization and non-distalization treatment groups in any of the six investigated variables (*P* > 0.05).

**Table 1:** Demographic comparison between treatment groups for gender distribution and age (mean ± SD)

**Group**	**Size (N)**	**Gender**	**N (%)**	**Age (Mean±SD)**
Group 1: Canine distalization	20	Female	11 (55)	25.30 ± 7.37
		Male	9 (45)	
Group 2: Alignment without canine distalization	10	Female	5 (50)	24.40 ± 8.39
		Male	5 (50)	
*P *value	-	0.79^⁕^	-	0.76^⁕⁕^

* Chi-squared test; ** Independent t-test.

**Table 2:** Descriptive statistics and *P*-values for alveolar ridge parameters by treatment group

**Variable**	**Group**	**Mean**	**Standard deviation**	**Maximum**	**Minimum**	***P*** ** value** **(Independent t-test)**
Alveolar ridge height (mm)	Distalization	17.72	2.43	22.42	13.24	0.83
	No distalization	17.52	2.41	21.16	13.89	
Ridge width at 3 mm from the edge of the alveolar crest (mm)	Distalization	5.21	1.06	7.21	3.30	0.31
	No distalization	4.82	0.81	6.23	3.90	
Ridge width at 6 mm from the edge of the alveolar crest (mm)	Distalization	4.97	1.42	7.65	2.85	0.59
	No distalization	5.25	1.06	6.60	3.75	
Bone density (Hounsfield units)	distalization	382.12	149.79	671.13	118.95	0.56
	No distalization	421.11	212.32	832.95	163.47	
Labial undercut depth (mm)	Distalization	1.35	0.47	2.35	0.42	0.53
	No distalization	1.49	0.80	2.50	0.15	

 In the canine distalization treatment group, the average width of the alveolar ridge at 3 mm from the edge of the alveolar crest was 0.24 mm more than the average width of the alveolar ridge at 6 mm from the edge of the alveolar crest (*P* = 0.20). In the group with non-distalization treatment, the average width of the alveolar ridge at 3 mm from the edge of the alveolar crest was 0.43 mm less than the average width of the ridge at 6 mm from the edge of the alveolar crest (*P* = 0.06). However, the difference between the average widths in the distalization and non-distalization groups was not significant (*P* > 0.05).

 Among the investigated correlation of variables with age, only alveolar ridge height in both treatment groups had a statistically significant relationship and a moderate inverse correlation with age (respectively with *P* < 0.001 and r = -0.58 in the distalization group and *P* < 0.01 and r = -0.73 in the non-distalization group). The width of the ridge at 3 and 6 mm from the edge of the alveolar crest, bone density, and depth of the labial undercut did not have a statistically significant relationship or a strong correlation with the age of the patients (*P* > 0.05, r < 0.3) in any of the two treatment groups.

## Discussion

 Restoring an edentulous area with an endosseous dental implant is among the most effective treatment options available for patients with congenitally missing lateral incisors. However, sufficient and appropriate bone dimensions are prerequisites for placing the implant in an ideal place.^4,10,11^ Considering that the presence of teeth with a healthy periodontium is necessary to maintain the width and height of the alveolar ridge, it is important to pay attention to the fact that in patients with congenitally missing lateral incisors, the ridge is narrow and reduced; as a result, it usually lacks suitable bone dimensions for placing the dental implant in the ideal place.^7^

 Orthodontic tooth movement includes bone resorption and formation, and tooth movement through the bone can affect bone dimensions in the edentulous area.^12^ The evaluation of the changes in alveolar ridge dimension in patients with maxillary lateral incisor agenesis after ridge development procedures by canine distalization has produced conflicting results. Some studies have reported minimal alveolar bone width loss,^9,10^ whereas others have shown significant decreases in alveolar ridge dimensions immediately after orthodontic treatment.^6-8^

 However, none of the available studies have directly compared patients with lateral incisor agenesis in the group with canine distalization versus the group without canine distalization, in terms of the amount of bone present at the site of the missing tooth. Instead, they have only compared the amount of bone present at the site of the missing lateral incisor before and after canine distalization in one group of patients who received this treatment and relied on plaster casts to do so, except in one study,^8^ which was not an accurate indicator of bone dimensions.^13^ These factors differentiate this study from others in this area as we divided the patients under investigation into two separate groups based on whether they received canine distalization treatment or not and attempted to investigate the effect of canine distalization on bone dimensions in CBCT images.

 The results of this study showed that in patients with congenitally missing maxillary lateral incisors aged 15–38 years, there was no significant difference in terms of alveolar ridge height, alveolar ridge width at 3 and 6 mm from the crest of the alveolus, depth of the buccal undercut, and alveolar density at the site of the missing lateral incisor between the group treated with distal movement of the canine during orthodontic treatment and the group without such movement. Therefore, it seems that movement of the canine along the edentulous ridge at the site of the missing lateral incisor cannot address the need for bone graft or ridge augmentation before implant placement. According to the findings of our study on alveolar bone density at the site of the missing tooth, in both the distalization and non-distalization treatment groups, the average density according to the Misch^14^ classification was in subtype D3, which is a favorable bone for implantation. For an ideal implant in the anterior region, the alveolar ridge width should be 6 mm and the height should be 12 mm.^15^ However, based on our study results in both the distalization and non-distalization treatment groups, the ridge width was < 6 mm on average at distances of 3 and 6 mm from the crest; therefore, it is not sufficient or suitable for implant placement in the ideal location, and bone grafting is required.

 Kokich^10^showed that after canine distalization, the dimensional changes of the alveolar ridge were minimal in the long term. However, in the article above, no explanation was given regarding the changes in bone dimensions immediately after orthodontic treatment. In a study by Nováčková et al,^9^ measurements taken on plaster casts of patients with congenital lateral incisor agenesis showed that during orthodontic treatment to open space for implants, the width and height of the alveolar bone decreased by 4% and 0.26 mm, respectively, immediately after distalization, compared to before treatment. The clinical significance of this was not meaningful, and the researchers concluded that the bone formed during orthodontic treatment was stable in both vertical and horizontal directions. However, in another study conducted by Uribe et al,^7^ the alveolar ridge width, height, and depth of the buccal undercut were measured on the plaster casts of patients with congenital lateral incisor agenesis before and after orthodontic treatment. Their results showed a significant decrease in alveolar bone width and height, as well as a doubling of the depth of the buccal undercut, in contrast to the results of a previous study. By examining the casts of 14 patients with congenital lateral missing teeth, Beyer et al^6^ also concluded that there was a significant decrease in bone volume in the edentulous ridge after orthodontic treatment. Despite the use of plaster casts for measurements in all three studies, the measurement methods for the width and height of the ridge were different, which could be one of the reasons for the varied results. On the other hand, due to the simultaneous measurement of hard and soft tissues in plaster models and the differences in the thickness of soft tissue in different people, and as a result, the impossibility of accurate measurement of available bone dimensions in this method, the use of plaster models to check the dimensions of the bone ridge does not seem to be reasonable and can be one of the reasons for the varied results of the studies.^13^

 CBCT scans display a patient’s hard tissue and do not exhibit distortion, magnification, and superimposition. Studies comparing CBCT and direct measurements have shown the high accuracy of CBCT scans in measuring the thickness and height of the buccal alveolar bone.^16,17^ According to literature research, only one study examined the effect of canine distalization treatment on alveolar ridge dimensions using CBCT. This study was performed based on CBCT scans before and after canine distalization in patients with unilateral missing lateral teeth, in which the canine erupted less than 2 mm from the central incisor. The results showed that during orthodontic treatment with space opening, the width of the alveolar ridge decreased by 17‒25%, and the depth of the buccal undercut increased; however, there was no significant change in the height of the alveolar ridge.^8^ The results of this study were similar to those of Uribe et al and Beyer and colleagues’^6,7^ studies in terms of width reduction of the ridge, but they were different in terms of no significant change in alveolar ridge height.

 In the present study, in the treated group with distalization, the mean width of the alveolar ridge decreased from 3 mm from the alveolar crest to 6 mm from the alveolar crest by 0.24 mm. In the non-distalization-treated group, the mean width of the alveolar ridge increased from 3 mm from the alveolar crest to 6 mm from the alveolar crest by 0.43 mm. However, Zhang et al^18^ demonstrated that the mean width of the alveolar ridge increased from the coronal to the apical region in patients with complete dentition in the maxillary lateral area. This difference between the distalization-treated group in our study and the patients examined in Zhang and colleagues’^18^ study can be attributed to the effect of orthodontic movement of the canine tooth along the alveolar ridge in the distalization-treated patients. Additionally, the mean age of patients in Zhang and colleagues’^18^ study was 45.25 years old, which differed significantly from the mean age group of patients in our study (mean age: 25 years); hence, the data obtained from the two studies cannot be confidently compared.

 This study also had several limitations; hence, the results should be interpreted with caution. This study was cross-sectional and only examined the association of independent and dependent variables and not their cause-and-effect relationship. In addition, CBCT scans at the beginning of sample treatment were not available; therefore, it was not possible to compare the initial dimensions of the ridge bone between the two groups and the dimensions and density of the alveolar bone at the beginning and end of orthodontic treatment for each sample. Another limitation of this study was the small number of patients in both groups, especially in the non-distalization treatment group. Therefore, it is recommended that future studies in this field be conducted prospectively, with equal and more sample sizes in groups and by preparing documents and CBCT scans at the beginning and end of treatment for the samples. Furthermore, the study’s failure to account for soft tissue thickness, a crucial factor in implant esthetics, represents another limitation.

## Conclusion

 In patients with congenitally missing maxillary lateral incisors whose orthodontic treatment plan included distalization of the canine tooth along the alveolar ridge to open the space for an endosseous dental implant, the average age, height of the alveolar ridge, and width of the ridge at 3 mm from the edge of the alveolar crest were higher than those in patients who did not undergo distalization. On the other hand, in the group of patients with non-distalization treatment, the average width of the ridge at 6 mm from the edge of the alveolar crest and the density and depth of the undercut were greater than those in the group of patients with canine distalization treatment; however, these differences were not statistically and clinically significant.

 Therefore, it seems that orthodontic space opening by canine distalization along the edentulous ridge does not develop sufficient bone dimensions for ideal dental implants; hence, this treatment cannot be considered a definitive alternative to bone grafting or ridge augmentation surgery for implant placement.

## Competing Interests

 The authors declare no conflicts of interest concerning the research, authorship, and/or publication of this article.

## Data Availability Statement

 The data that support the findings of this study are available upon request from the corresponding author.

## Ethical Approval

 The study protocol of the present study was approved by the Ethics Committee of the Mashhad University of Medical Science (IR.MUMS.DENTISTRY.REC.1400.038).
